# Out in the open: behavior’s effect on predation risk and thermoregulation by aposematic caterpillars

**DOI:** 10.1093/beheco/araa048

**Published:** 2020-05-20

**Authors:** Matthew E Nielsen, Johanna Mappes

**Affiliations:** 1 Department of Biological and Environmental Sciences, University of Jyväskylä, Survontie 9 C, Jyväskylä, Finland; 2 Department of Zoology, Stockholm University, Svante Arrhenius väg 18b, Stockholm, Sweden

**Keywords:** aposematism, *Arctia plantaginis*, color, microhabitat preference, *Parus major*, thermoregulation

## Abstract

Warning coloration should be under strong stabilizing selection but often displays considerable intraspecific variation. Opposing selection on color by predators and temperature is one potential explanation for this seeming paradox. Despite the importance of behavior for both predator avoidance and thermoregulation, its role in mediating selection by predators and temperature on warning coloration has received little attention. Wood tiger moth caterpillars, *Arctia plantaginis*, have aposematic coloration, an orange patch on the black body. The size of the orange patch varies considerably: individuals with larger patches are safer from predators, but having a small patch is beneficial in cool environments. We investigated microhabitat preference by these caterpillars and how it interacted with their coloration. We expected caterpillar behavior to reflect a balance between spending time exposed to maximize basking and spending time concealed to avoid detection by predators. Instead, we found that caterpillars preferred exposed locations regardless of their coloration. Whether caterpillars were exposed or concealed had a strong effect on both temperature and predation risk, but caterpillars in exposed locations were both much warmer and less likely to be attacked by a bird predator (great tits, *Parus major*). This shared optimum may explain why we observed so little variation in caterpillar behavior and demonstrates the important effects of behavior on multiple functions of coloration.

## Teaser

Predation and temperature are two major forces that influence survival. We found that wood tiger moth caterpillars frequently bask in exposed locations, although this should also expose them to predators. Nevertheless, in our experiments, exposed caterpillars both stayed warmer and were attacked less by birds compared with hidden caterpillars. Warning coloration, thus, protects these caterpillars from birds, but more so if these caterpillars are easily found.

## INTRODUCTION

Warning colors are a classic example of traits that should be under strong stabilizing selection yet often display large amounts of intraspecific variation (Joron and Mallet 1998; Mappes et al. 2005; Stevens and Ruxton 2012). One of the main proposed mechanisms underlying variation in aposematic coloration is opposing selection due to the many different functions of color combined with variation in the strength of these sources of selection (Briolat et al. 2019). In particular, coloration has a key role in thermoregulation, particularly by ectotherms. To maximize fitness, ectotherms need to stay warm without becoming too hot (Huey and Kingsolver 1989). Color determines how efficiently ectotherms absorb sunlight, with dark colors favored in cool environments to help stay warm and light colors favored in hot environments (Clusella Trullas et al. 2007). At the same time, darker colors, although favored by cool environments, can make less effective warming signals, particularly if they reduce internal contrast or contrast with their background (Prudic et al. 2006; Lindstedt et al. 2009; Hegna et al. 2013; Arenas et al. 2015).

Both warning signaling and thermoregulation depend heavily on behavior, particularly microhabitat selection, which has received surprisingly little attention in studies of these multiple functions of color. A highly concealed organism will be harder for predators to detect, regardless of coloration, so aposematism has been argued to be more likely to evolve in behaviorally exposed organisms (Speed and Ruxton 2005; Grant 2007). Even then, aposematic organisms can still face some risk of predation from naïve predators and predators, which can overcome their defenses (Endler and Mappes 2004; Mappes et al. 2014). Thus, aposematic organisms should still prefer concealed microhabitats unless the exposed locations offer some other benefit, such as food availability (Speed et al. 2010). At the same time, microhabitat selection greatly affects many factors that determine an ectotherm’s body temperature, including exposure to solar radiation (Stevenson 1985; Muñoz et al. 2014). Thus, at least in cool environments, microhabitat preference should experience opposing selection: thermoregulation should favor basking in an exposed, sunny microhabitat, but these locations should be associated with a greater risk of predation, even for aposematic animals.

To test how behavior alters both thermoregulation and aposematic signaling, we studied the caterpillars of *Arctia plantaginis* (Linneaus; formerly *Parasemia plantaginis*; Rönkä et al. 2016). The aposematic function of the adult moth’s color pattern has been extensively studied (e.g., Lindstedt et al. 2011; Nokelainen et al. 2012; 2014), and the degree of melanization in adults has also been shown to affect their body temperature (Hegna et al. 2013). A similar relationship appears to exist in the caterpillars. *Arctia plantaginis* caterpillars are black with an orange band, but there is both genetic and plastic variation in the width of this band (Ojala et al. 2007; Lindstedt et al. 2009, 2010, 2016; Galarza et al. 2019). Predatory birds (specifically *Parus major*) learn to avoid caterpillars with broader orange bands more quickly (Lindstedt et al. 2008), likely due to the greater conspicuousness of this pattern (Mappes et al. 2014). On the other hand, caterpillars with smaller orange bands and correspondingly more black coloration grow faster in cool temperatures, likely because of improved thermoregulatory ability (Lindstedt et al. 2009). Caterpillar behavior could heavily influence the optimization of predation risk and thermoregulation, but the behavior of these caterpillars—like most caterpillars—has received little attention. Nevertheless, a previous study suggests that color pattern may affect the basking behavior of these caterpillars under cool temperatures (Lindstedt et al. 2009).

We started by investigating the behavior of *A. plantaginis*, specifically the caterpillars’ preference for exposed or concealed microhabitats. In our studies, we used caterpillars from central Finland, where staying warm should be a priority for growth, but predation rates are also relatively high (at least for adult moths; Nokelainen 2014). Thus, we expected behavior to reflect a balance between minimizing exposure to potential predators and maintaining a warm body temperature. Then, we investigated the effects of color pattern and microhabitat preference on temperature and predator avoidance using wild great tits, *P. major*, as a model predator*. Parus major* is a common generalist feeder and potential predator of *A. plantaginis* in the wild, which can easily be trained for a variety of foraging tasks in the lab and has been used in previous laboratory experiments on *A. plantaginis* predation (Lindstedt et al. 2008, 2011). For both traits, we predicted opposing selection by thermoregulation and predator avoidance: smaller orange bands (thus, more melanized caterpillars) would be favored for thermoregulation but selected against by predation and, similarly, exposed locations would be favored for thermoregulation but selected against by predation.

## METHODS

### Rearing of caterpillars and general procedures

*Arctia plantaginis* caterpillars used in experiments came from a laboratory stock population originally derived from wild females caught in Central Finland in 2012 and supplemented annually with new wild-caught individuals. The stock was maintained in a greenhouse at the University of Jyväskylä on a mixed diet of lettuce (*Lactuca sativa* var. *crispa*) and dandelion (*Taraxacum* sp.). Experiments were conducted in 2015, at which point the lab stock had been maintained for eight generations. This species usually has one generation per year in the wild and typically overwinters as third or fourth instar larva. Under laboratory conditions, *A. plantaginis* moths can produce three generations per year and the third generation overwinters. Temperatures in the greenhouse followed the outdoor temperatures and varied between 20 and 30 °C during the day (~20 h) and decreased to 15–20 °C during the night (~4 h). These temperatures correspond to average temperatures in July in Jyväskylä (data available at the Finnish meteorological institute). See Lindstedt et al. 2016 for more details on general caterpillar rearing. To quantify the length of the orange band, we counted the number of body segments with at least some orange hairs on them (Ojala et al. 2007). For experiments, caterpillars of the appropriate orange band length were haphazardly selected from among 14 families, with some caterpillars reused across live-caterpillar experiments due to limited availability of individuals with certain orange band lengths.

## Short-term preference for exposed versus concealed locations

To determine the temperature threshold at which caterpillars switched from preferring exposed to concealed locations, we placed single caterpillars (*n* = 50) of varying orange band lengths (3–7 segments) under a halogen light (400 W, 8550 lm, 2800 K color temperature, manufactured by CRX) and measured the time until the caterpillars sought shade under a host and the temperature at which they did so. During the experiment, room temperature was held at ~15 °C. At the start of each trial, we placed a caterpillar on a potted dandelion (*Taraxacum* sp.), which had been collected that day from outside (individual plants were reused for multiple tests). After the caterpillar stopped moving, we turned on a 400-W halogen lampplaced 40 cm directly over the caterpillar and plant, gently repositioning any leaves that were initially shading the caterpillar (this never led to immediate movement). Each time the caterpillar began moving, we recorded the time elapsed, took a thermal image of the caterpillar using an infrared camera (FLIR C2, FLIR Systems), and recorded shade temperature using a thermocouple shaded by a leaf. When the caterpillar stopped moving in a location that was at least 20% shaded, we considered the prior movement thermoregulatory behavior and used the associated data for our analysis. After excluding trials that did not occur properly (see Supplementary Methods for more details), final sample sizes were 45, 44, and 45 for time, ambient temperature, and body temperature respectively.

To estimate the body temperature of the caterpillar from each image, we first converted the image to linear-temperature grayscale using FLIR Tools (v6.3, FLIR Systems). Then, we measured the temperature by outlining the caterpillar by hand in imageJ (v1.51; Schneider et al. 2012) and computing the mean temperature. We evaluated the replicability of this approach by having another person remeasure 10 randomly selected images. These two measurements were highly correlated (*r* = 0.998), indicating the precision and consistency of this approach. The repeated measurements differed significantly by a mean of 0.53 °C (paired *t*-test, standard deviation = 0.23, *t*_9_*=* 7.14, *P =* 5.4 × 10^–5^), so this approach should be accurate within 1 °C. We tested whether the body temperature, shade temperature, and time from the start of heating at which the caterpillar sought shade were affected by the length of the caterpillar’s orange band using separate linear mixed-effects models with the individual plant used in the trial as a random effect. Caterpillar mass (measured posttrial with 0.1 mg precision) was initially included as an additional fixed effect; however, it had no significant effect on the results for any response variable (*P* > 0.2), so it was excluded from the final analysis. All statistical analyses were conducted in R (v3.4.0; R Core Team 2018). For this analysis, we used the lme function in the nlme package (Pinheiro et al. 2018) and *P*-values were generated using likelihood ratio tests between models with and without each fixed effect.

## Long-term preference for exposed versus concealed locations

To test overall preference of caterpillars for exposed versus concealed positions over longer time periods, including whether color pattern affected this preference, we placed caterpillars (*n* = 36) in sets of three (one each of band lengths 4, 5, and 6 segments) on host plants (*n* = 12), and left them there under less intense heat to observe how much time they spent exposed during the day. As before, recently potted dandelions (*Taraxacum* sp.) were used as hosts; however, in this case, the dandelion was enclosed in a cylinder of clear acrylic to prevent escape and the leaves were trimmed to fit. The evening before the trial, the caterpillars were placed on each plant and allowed to adjust to the chambers overnight. Size of the orange patch size was used to distinguish each of the three individuals per plant during observations. The next morning, at 9 AM, we turned on two 400-W halogen lights ~75 cm over the table. At this time, 24 out of 36 caterpillars were in exposed positions. Over the next 9 h, we observed the position of each caterpillar every 30 min, noting whether the caterpillar was fully exposed or at least partially (20%) hidden. Of necessity, enclosures varied in exact distance from these lights, producing a range of mean surface temperatures from 14.5 to 27.3 °C among the enclosures over the course of the experiment (mean temperature of each enclosure in overhead thermal images taken every 30 min). Caterpillars that showed signs of building a shelter in which to molt or otherwise starting to molt were excluded from analysis from that observation onward (101 observations were excluded out of 708 in total; 11 caterpillars had observations excluded, and only one individual was fully excluded).

To test for an effect of orange band length on preference for exposed versus hidden positions, we used a generalized linear mixed model with a binomial distribution. We used whether the caterpillar was observed exposed as our dependent variable, band length and enclosure surface temperature as fixed effects, and individual nested within pot as a random effect. Models were fit using the glmer function in the lme4 package (Bates et al. 2015) in R, and a *P*-value was generated using a likelihood ratio test between models with and without each fixed effect.

## Effect of color and position on caterpillar temperature

To test how exposure and orange band length affect the body temperature of caterpillars, we measured the temperature of caterpillars (*n* = 48) when placed outside under sunny conditions either with or without artificial shading. We tested caterpillars with orange band lengths from 4 to 7 segments, 12 of each size. Caterpillars were tethered to a piece of cardboard with 5 cm between individuals using thin string to immobilize them. Caterpillars of each signal size were evenly divided between two sides but otherwise placed randomly. Over the course of the experiment, three individuals escaped and were thus excluded from analysis. We conducted the experiment in Jyväskylä (62.230°N, 25.744°E) on 25 September 2015. On this day, we placed the tethered caterpillars outside at 2:38 PM and started the experiment when, at 3:31 PM, an extended period of continuous sunlight began. After 28 min of unobscured sunlight, half of the array was shaded by a piece of cardboard ~30 cm above the ground. During the period of unobscured sunlight, we periodically recorded thermal images of the caterpillars, ultimately using the last pictures taken for each condition during full sunlight (after 25 min without shade and after 20 min with half shaded) since these should represent near-equilibrium temperatures. Ambient temperatures (recorded using a shaded thermocouple) during these two measurements were within 0.5 °C of each other and, thus, were not factored into the analysis.

To analyze these images, we used a similar method to the short-term preference experiment; however, resolution and focal limitations of the thermal camera prevented precise identification of the edges of each caterpillar. Instead, we estimated each caterpillar’s temperature by using ImageJ to find mean temperature of a 6-pixel diameter circle centered on the warmest point in each caterpillar. We also estimated the temperature of a circle at the center of the cardboard background in each image to compare its temperature in the shade versus sun treatments (Supplementary Methods). We analyzed the effects of caterpillar orange band length, whether the caterpillar was exposed or shaded, and their interaction using a linear mixed-effects model. We included caterpillar mass as an additional fixed effect and the identity of each caterpillar as a random effect. We also allowed variance to depend on shading treatment. We used the lme function in the nlme package for this analysis and *P*-values were generated using likelihood ratio tests between the full model and models without each fixed effect. To further examine the large effect of the shading treatment that we found, we also performed an additional likelihood ratio test comparing models with no interaction term and with or without shading treatment.

## Effect of color and position on predation risk

To test how exposure and orange band length affected relative predation risk, we conducted a laboratory experiment using dead caterpillars and wild-caught *P. major* (great tits). Forty-eight birds were trapped between 6 August and 8 December 2015 and kept for up to 2 weeks in captivity for trials with *A. plantaginis,* and 16 birds were trapped between 14 February and 1 March for trials with mealworms. In captivity, they were housed individually in plywood cages under an 11:13 h (light:dark) photoperiod and fed with sunflower seeds and vitamin-enriched tall ad libidum. Prior to the experiment, birds had no previous experience in captivity with *A. plantaginis* (whether they had encountered *A. plantaginis* in the wild is unknown but unlikely because there are no known wild population of *A. plantaginis* in the vicinity of Konnevesi research station).

Each bird was given an array of 12 caterpillars that were all either exposed or concealed but varied within array in orange band length from 4 to 6. This created two overall treatments (exposed vs. concealed) while also creating within-treatment variation in warning coloration. Each array contained 12 dead *A. plantaginis* caterpillars (killed by freezing but thawed before use): 4 each of band length 4, 5, and 6. Each caterpillar was also weighed before the trial. Arrays were presented to birds in a 21.0- × 29.7-mm cardboard box with a foam insert covered in brown paper. We filled this box with a 2–3-cm deep layer of dried, dead leaves, predominantly *Betula pendula* (silver birch). Each caterpillar was pinned in the box using a black-painted insect pin, 2–3 cm from the edge and 8–9 cm from each other, with individuals assigned to specific locations randomly. Depending on treatment, we pinned all caterpillars either below (concealed) or on top of (exposed) the layer of leaves. To ensure we had enough caterpillars for all replicates of the experiments, some trials were conducted using caterpillars descended from *A. plantaginis* originally collected in Estonia (proportion of caterpillars attacked during each trial did not differ significantly between Finnish and Estonian populations and, thus, were pooled for all analyses; *t*-test, *t*_33_ = 0.275, *P* = 0.785). We also repeated both the hidden and exposed treatments using arrays of 12 mealworms instead of *A. plantaginis* caterpillars to test the effect of concealment on palatable and nonwarningly colored prey.

Trials were conducted in a separate wooden box (50-cm wide × 50-cm deep × 67-cm high) with a one-way mirror to allow observation and recording (Supplementary Methods). Before each trial, each bird was food deprived for 2 h total, 90 min in its home box and 30 min acclimating to the trial box. At that time, the lights in the box were briefly turned off and the array placed in the box. Birds that did not begin foraging (as indicated by interacting with a caterpillar) within 45 min after the lights were turned back on were excluded from the study as were birds that foraged for less than 30 min total within 115 min with the box. We ultimately excluded 12 birds in this way, evenly divided between treatments. Once foraging began, the trial continued until either the bird had attacked all caterpillars or 30 min of foraging had elapsed (excluding any breaks 5 min or longer spent not interacting with caterpillars or the box). During the trial, we noted which caterpillars were attacked, specifically, if the caterpillar was pulled off its pin or, in a few cases, consumed while on the pin. We also recorded when the attack occurred to provide information about the order of attack. At the end of the trial, we confirmed which caterpillars were attacked by checking which caterpillars remained on their pins. Except for the first two birds tested, we also estimated the total mass of caterpillar the bird ate by weighing the caterpillar remains of the attacked caterpillars and subtracting from their original weight. In one case, the estimated mass eaten was slightly negative (likely due to measurement error) and was set to 0 because it indicated that the bird had eaten none of the caterpillar despite removing them from the pin. Overall, we successfully tested 36 birds, 18 each with concealed or exposed caterpillars, plus 16 more birds with mealworms, 8 each concealed and exposed.

We tested how caterpillar exposure affected whether a caterpillar was attacked during a trial using a generalized mixed model with a binomial distribution, with exposure treatment as a fixed effect and individual bird as a random effect. To account for rejection of prey after attack, we also tested the effect of caterpillar exposure on the total mass consumed by each bird using a linear model with exposure as a fixed effect. We repeated both analyses separately for the mealworms. To analyze the effects of color on predation risk, we considered only the first six attacks by each bird (or fewer if the bird attacked less than six caterpillars during the trial) because each bird only had access to three caterpillars of each band length, so the distribution of attacks would necessarily converge toward an equal attack rate on all band lengths if all attacks were considered. We then fit a generalized mixed model using a binomial distribution for whether or not each caterpillar was one of the first six attacked. Exposure treatment, orange band length (as a factor), mass, and row in the array (indicating proximity to the bird’s initial perch) were used as fixed effects, whereas individual bird was a random effect. The mixed models were fit using the glmer function in the lme4 package in R and *P*-values were generated using a likelihood ratio test between models with and without each fixed effect. All second- and third-degree interactions among fixed effects were nonsignificant (*P* > 0.2) and excluded from the final analysis. We performed a Tukey’s post hoc test on the final model for the differences among different orange band lengths using the package emmeans (Lenth 2018) and generating *P*-values from the resulting *z*-scores.

## RESULTS

### Effect of color on behavioral exposure—short term

**Figure 1:**
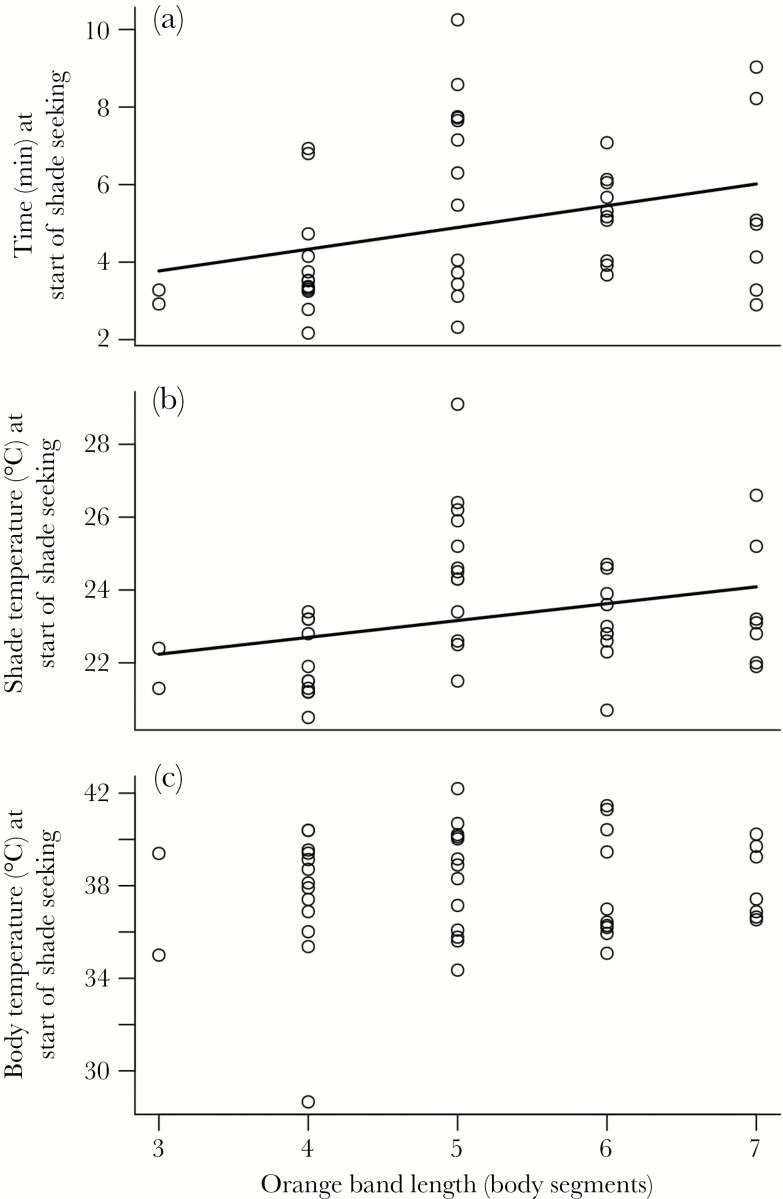
Effects of caterpillar orange band length (in number of segments) on the start of shade-seeking behavior when heated by an overhead halogen light. Lines represent significant (*P* < 0.05) fixed effects of band length on the response variable in a mixed model. (a) Time (minutes) from start of experiment, *n* = 45. (b) Surface temperature (degree Celsius) of shaded ground, *n* =44. (c) Body temperature (degree Celsius) of caterpillar, *n* = 45.

We successfully tested 45 caterpillars for conditions under which they sought at least partial shade. Caterpillars with larger orange bands sought shade significantly later than those with smaller orange bands (β *=* 0.56 min/segment, standard error [SE] *=* 0.24, = 4.80, *P* = 0.028; Figure 1a) and they also did so at higher shade temperatures (β *=* 0.46 °C/segment, SE *=* 0.22, = 4.29, *P* = 0.038; Figure 1b). Band length did not, however, significantly affect the body temperature at which caterpillars began seeking shade (β *=* 0.18 °C/segment, SE = 0.33, = 0.329, *P* = 0.57; Figure 1c), which was quite high overall with a mean of 37.9 °C.

## Effect of color on behavioral exposure—long term

We observed 35 caterpillars over a 9-h period. During the vast majority (91.8%) of our observations, caterpillars were fully exposed to the light, spending only 8.2% of their time fully or partially concealed. Although caterpillars chose concealed locations infrequently, orange band length nevertheless affected how often this occurred. Caterpillars with larger orange bands were significantly more likely to be observed fully exposed (β *=* 0.836 ± 0.276, = 8.43, *P* = 0.004). Specifically, caterpillars with four orange segments were exposed 84.4% of the time, five orange segments 95.0% of the time, and six orange segments 95.7% of the time. Caterpillars were also significantly more likely to be observed fully exposed when the enclosure was warmer (β *=* 0.233 ± 0.066, = 12.09, *P* = 0.0005).

## Effect of color and position on temperature

**Figure 2:**
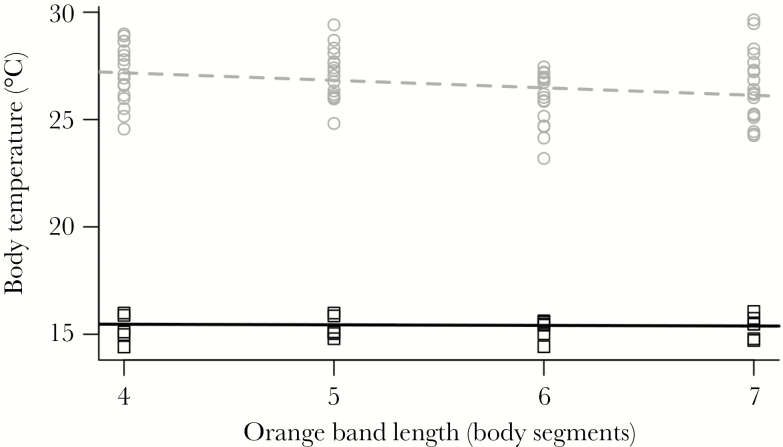
Body temperature of caterpillars with varying orange band lengths when placed outdoors either in full sun or shade. Gray circles and dashed line represent caterpillars in full sun; black squares and solid line represent caterpillars in shade. Lines show interacting fixed effects from a mixed model (*P* = 0.0365). *n* = 90 observations of 45 caterpillars.

We measured the temperature of 45 caterpillars under sunny conditions twice, once with all in full sun the other with half in full sun and half shaded. We found a significant interaction between the effects of orange band length and shade treatment on temperature (Table 1; Figure 2); however, the relative effect sizes of these two factors differed dramatically. Shading had a large overall effect on caterpillar temperature, cooling caterpillars by 10.7 °C averaged across orange band lengths ( = 142.3, *P* < 0.0001). This difference is only partially explained by the effect of shading on the caterpillar’s microhabitat (the temperature of the cardboard background differed by 6.6 °C between these treatments). Orange band length, on the other hand, had only a small effect on temperature when in the sun (−0.349 °C/orange segment) and practically no effect when shaded (−0.027 °C/orange segment). Caterpillar mass ranged from 0.108 to 0.307 g (mean 0.195 g), and body temperature increased significantly with mass by 9.63 °C/g (Table 1).

**Table 1:** Effects of orange band length, position (sun or shade), and mass on temperature (degree Celsius) of *A. plantaginis* caterpillars

	Coeff.	SE	df	*χ* ^2^	*P*
Orange band length	−0.0270	0.090	1	—	—
Position^a^	13.00	0.879	1	—	—
Mass (g)	9.63	2.7	1	12.52	0.0004
Orange band length × position^a^	−0.322	0.155	1	4.37	0.0365

df, degrees of freedom.

^a^Difference between sun and shade.

## Effect of color and position on predation risk

**Figure 3:**
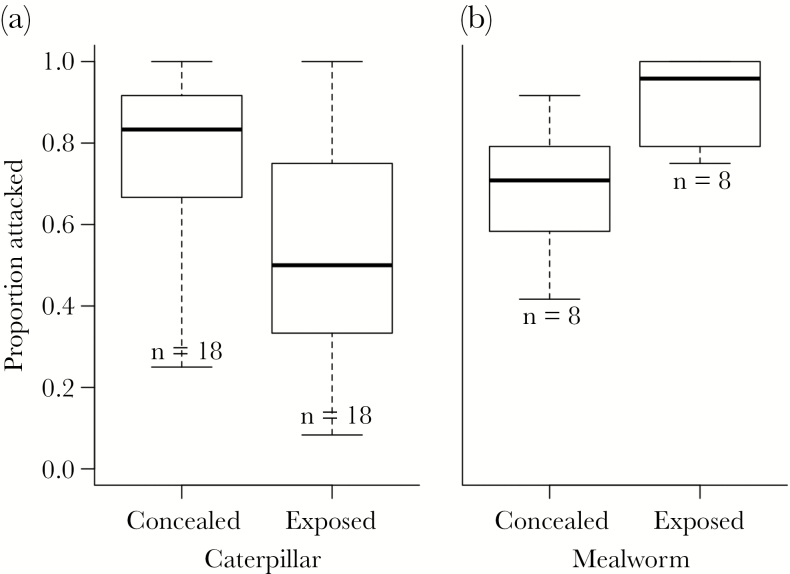
Boxplot of proportion of (a) *A. plantaginis* caterpillars or (b) mealworms attacked by *P. major* during foraging trials. Each bird had access to either 12 dead caterpillars or mealworms, all of which were pinned in place either exposed on top of or concealed beneath a layer of dead leaves.

During 30 min of foraging, *P. major* attacked a smaller proportion of *A. plantaginis* caterpillars in the exposed than concealed treatment (β *=* −1.27 ± 0.48, = 6.53, *P* = 0.011; Figure 3). This corresponds to a mean of 2.61 fewer caterpillars (out of 12) attacked when exposed. From the attacked caterpillars, the birds ate, on average, 0.372 g less *A. plantaginis* caterpillar in the exposed than concealed treatment (SE *=* 0.124 g, *t*_32_ = −3.00, *P =* 0.005). This mass is equivalent to 2.55 caterpillars (mean mass of attacked caterpillars was 0.146 g, SE = 0.0028), indicating that most of the additional caterpillars attacked when concealed were also consumed. In contrast, when foraging on mealworms, birds attacked a greater proportion of individuals in the exposed than concealed treatment (β *=* 1.54 ± 0.50, = 8.63, *P* = 0.003; Figure 3), corresponding to a mean of 2.62 more mealworms attacked when exposed. The birds did not, however, eat significantly more mealworms (0.123 g, SE = 0.102 g, *t*_14_ = 1.20, *P* = 0.25).

**Figure 4:**
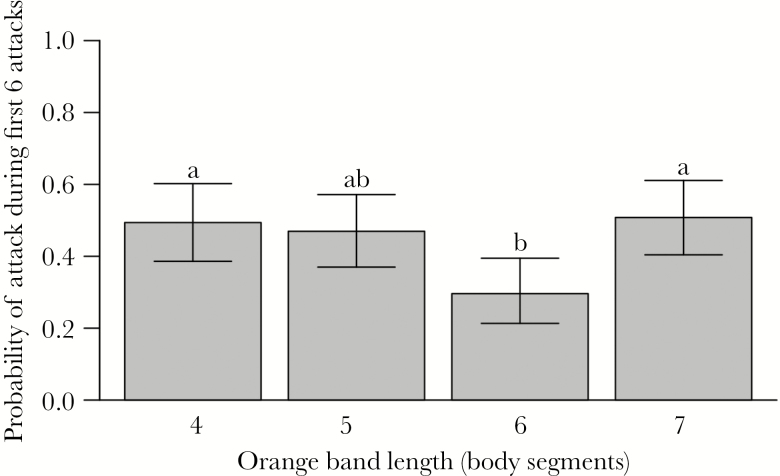
Probability of *A. plantaginis* caterpillars with varying orange band length being among the first six attacked by *P. major* in an array of 12 caterpillars (three of each band length). Bars represent 95% confidence interval, and lettering indicates significantly differing band lengths in a Tukey’s post hoc test (*n* = 34 birds).

To assess how color affected risk of predation, we focused on the first six caterpillars attacked by each bird. Larger caterpillars were more likely to be attacked, as were caterpillars closer to the bird’s perch (Table 2). Most importantly, orange band length had a significant effect on risk of predation (Table 2; Figure 4); specifically, caterpillars with an orange band 6 segments long were least likely to be among the first six caterpillars attacked and significantly less likely to be attacked than caterpillars with an orange band 4 or 7 segments long according to post hoc tests. Given that we limited our analysis to the first six caterpillars attacked, it is unsurprising that exposure had no significant independent effect on attack risk; however, it also did not significantly interact with orange band length ( = 1.08, *P* = 0.78).

**Table 2:** Effects of exposure treatment, orange band length, mass, and position relative to bird’s perch (rows away) on whether *A. plantaginis* caterpillars were among the first six caterpillars attacked in an array of 12 by *P. major* in laboratory trials

	Coeff.	SE	df	*χ* ^2^	*P*
Treatment^a^	−0.324	0.213	1	2.33	0.127
Orange band length^b^	—	—	3	11.28	0.0103
5 body segments	−0.097	0.305		—	—
6 body segments	−0.841	0.326		—	—
7 body segments	0.056	0.321		—	—
Mass (g)	8.75	2.43	1	13.55	0.0002
Distance from perch (rows)	−0.980	0.138	1	57.35	<0.0001

^a^Difference between exposed and concealed treatments.

^b^Analyzed as a factor. *χ*^2^ and *P*-values tested across all levels, coefficients given separately for each level compared with a length of four body segments as a baseline. See Figure 4 for more details.

## DISCUSSION

Coloration of aposematic animals provides an example of opposing selection promoting trait variation: trade-offs between warning signal efficacy and thermoregulatory ability (as well as other functions) can increase variation in a trait that should otherwise be highly conserved (Briolat et al. 2019). We predicted a similar opposing selection on microhabitat choice in *A. plantaginis,* an aposematic caterpillar, so we tested how color pattern and behavior interact with body temperature and predation risk in this species. We found that, although the effects of these two traits were not entirely independent, behavior had a greater effect on both sources of selection.

Contrary to our initial expectations, however, we found no evidence of opposing selection on microhabitat preference in *A. plantaginis* caterpillars. Individuals placed in exposed positions were both much warmer and less likely to be attacked by *P. major* in our experiments. This parallel selection by both predators and temperature favoring caterpillars in exposed locations helps explain the strong preference for exposed positions displayed by the caterpillars in our behavioral experiments. Over 9 h of observations under moderately warm thermal conditions, caterpillars spent the vast majority of their time in the open. This behavior suggests that these caterpillars rely on their primary and secondary defense strategies (coloration, hairiness, and chemical defense) for predator avoidance. Our findings support “a classic view of aposematism” in which conspicuousness reduces the likelihood of recognition errors because predators can detect conspicuous prey at a greater distance and, thus, avoid them more reliably (Guilford 1986). There has, however, been surprisingly few systematic observations of microhabitat selection by aposematic prey in this regard (but see Tabadkani and Nozari 2014; Rößler et al. 2019).

From a thermal perspective, caterpillars did not seek shade until heated to a surprisingly high mean body temperature of 37.9 °C. This body temperature is comparable to and, in some cases, exceeds the body temperature at which other species of caterpillar from much warmer environments begin heat-avoidance behavior (Sherman and Watt 1973; Nielsen et al. 2018). This observation fits with a general trend that the upper thermal limits of both insects and ectotherms decrease little with latitude (Addo-Bediako et al. 2000; Sunday et al. 2014). For high-latitude arthropods, it can be important to capitalize on brief warm, sunny periods to maximize growth (Kukal et al. 1988; Bennett et al. 1999; Birkemoe and Leinaas 2000).

On the other hand, the preference for exposure should represent a risk for animals with warning colors (Endler and Mappes 2004; Mappes et al. 2014). This risk should be especially great for aposematic animals like *A. plantaginis* that are only somewhat distasteful, so experienced predators will still eat them in the absence of preferred prey as occurred in our experiment. Nevertheless, we found, under laboratory conditions at least, that potential avian predators (wild-caught, adult *P. major*) attacked fewer *A. plantaginis* caterpillars when the caterpillars were exposed than when they were concealed under dead leaves, despite the latter requiring more effort to locate the caterpillars. The opposite pattern occurred with palatable, nonaposematic mealworms. The greater risk to concealed *A. plantaginis* is reinforced by the fact that birds attacked similar proportions of concealed caterpillars and concealed mealworms during the respective experiments, despite how *P. major* strongly prefers mealworms. Birds may have attacked more hidden caterpillars because the leaves obscured the warning signals of the caterpillars. If this hypothesis explained our results, however, we would have expected concealment to alter the effect of orange band length on attack risk, which we did not observe. Instead, the greater effort required to find concealed caterpillars could explain the greater willingness of *P. major* to attack them. Birds were frequently observed digging through leaves even in the exposed treatment when nothing was present there. In the exposed treatment, the birds may have initially ignored the exposed caterpillars to search for the potentially better prey that could have existed under leaves in the wild. In the concealed treatment, on the other hand, by the time the birds found the caterpillars hidden under the leaves, there were no additional locations to search, so the birds may have been more willing to accept them as prey. Notably, this digging behavior was performed by wild-caught birds without training. Indeed, great tits have a greater propensity than other tits for feeding on the ground (Gosler and Clement 2007). Regardless of the underlying cause, the lower attack risk for exposed caterpillars combined with their warmer temperature helps explain the strong behavioral preference we observed in these caterpillars for open, exposed positions. Based on these results, we would predict opposing selection by predation and temperature on microhabitat preference in warm environments instead of cool ones, but the trade-off may not occur in that context either. *Battus philenor*, a desert-dwelling aposematic caterpillar, remains fully exposed while still avoiding high temperatures (Nice and Fordyce 2006; Nielsen and Papaj 2017).

Variation in color, on the other hand, had much weaker effects than behavior on both body temperature and predation risk. For temperature, the effect of orange band length on body temperature depended heavily on the position of the caterpillar and, thus, its behavior: caterpillars with smaller orange bands (more melanin) were warmer as expected when exposed to the sun, but band length had little effect in the shade. Color and other aspects of morphology are generally predicted to have a weaker effect on temperature than behavior (Stevenson 1985), and the effect of color on temperature has been shown to depend on behavior in a wide range of insects (e.g., Kingsolver 1987; Nielsen and Papaj 2017). Despite its smaller effect, color pattern did alter thermoregulatory behavior in *A. plantaginis.* The body temperature at which shade-seeking began did not change with band length; however, caterpillars with shorter orange bands (more melanic caterpillars) started shade-seeking behavior sooner and at lower environmental temperatures. These results indicate that the caterpillars’ internal physiology and response to body temperature do not change with color pattern and, instead, color pattern alters behavior by changing light absorption and, thus, heating rate (Nielsen et al. 2018). This ability of body color to affect thermoregulatory behavior by changing body temperature has also been demonstrated in a range of insects (e.g., Kingsolver 1987; Karpestam et al. 2012; Nielsen et al. 2018).

We confirmed effect of color pattern on predation risk by comparing the first six caterpillars attacked by *P. major* during experiments. Caterpillar with an orange band 6 body segments long were less likely to be attacked, regardless of whether they were hidden or exposed. This is longer than the average signal size of *A. plantaginis* (4.7 segments) but not the largest possible (7 segments). Previous work on *A. plantaginis* indicates that larger orange bands (>5 segments) are more effective warning signals than short bands (<4 segments; Lindstedt et al. 2008, Mappes et al. 2014). We add to this that warning signal effectiveness might also decrease for the longest bands (7 segments). The small black content of these caterpillars could reduce their internal contrast, which, in turn, could reduce the effectiveness of the caterpillars’ warning signals (Aronsson and Gamberale-Stille 2013; Barnett et al 2016; although see Aronsson and Gamberale-Stille 2009). Regardless of the exact relationship between band length and predation, the orange band lengths least likely to be attacked differed from those which were warmest, indicating the expected opposing selection on color pattern from temperature and predation.

Ultimately, we found the expected opposing selection on one trait (color pattern) but not another (microhabitat preference). The presence or absence of opposing sources of selection could help explain the differing levels of phenotypic variation observed in these traits. Color pattern in both adult and larval *A. plantaginis* varies extensively due to both genetic and plastic factors both within and between populations (Ojala et al. 2007; Lindstedt et al. 2009, 2016; Hegna et al. 2013, 2015), and opposing selection from a variety of sources, including sexual selection, initial detectability, and thermoregulation, has been argued to enable persistence of this variation (Lindstedt et al. 2008, 2009; Nokelainen et al. 2012; Hegna et al. 2013; Mappes et al. 2014). The behavior of *A. plantaginis*, on the other hand, has received little attention, particularly in the caterpillars, but they generally display little activity, at least under laboratory conditions. The minimal variation we find in preferred microhabitat, and perhaps other aspects of their behavior, could be caused by having a single preferred microhabitat favored by multiple sources of selection removing the need for temporal or between-individual variation in habitat preference.

An important general benefit of aposematism is the opportunity to occupy microhabitats that are otherwise highly vulnerable to predation (Speed et al. 2010). Here, we find that this effect in *A. plantaginis* is stronger than anticipated despite the fact that the species is only unpalatable and hairy rather than truly toxic. This benefit of aposematism effectively negates the opposing selection faced by cryptic organisms between behaviors that maximize resource use (including favorable thermal environments) and minimize predator exposure (Speed and Ruxton 2005). Multiple other aposematic animals behave in ways that expose them more to predators than comparable cryptic animals (Pinheiro 1996, 2007; Rudh et al. 2013; Willink et al. 2013; Tabadkani and Nozari 2014; Valkonen et al. 2014; Rößler et al. 2019). Based on our results, we predict that aposematic organisms may frequently use different microhabitats from cryptic organisms and may show reduced behavioral variation at least in terms of habitat preference or antipredator behavior. At the same time, if an aposematic species is thus specialized for an exposed microhabitat due to parallel selection from multiple sources, they may be more vulnerable to any environmental changes, such as climate change, which reduce their fitness in that habitat. Future research could test for selection to prefer exposed microhabitats in other aposematic species and consider additional sources of selection, which may benefit exposure, such as sexual selection and foraging.

Regardless, we have shown for an aposematic species that, when facing wild predators, they are less likely to be attacked when exposed. Combined with the thermal benefits of exposure, the greater safety of caterpillars when exposed helps explain their minimal behavioral variation. Caterpillars rarely occupied concealed positions except at extreme temperatures. Although variation in color pattern had smaller direct effects than exposure on temperature and predation risk in our experiments, color pattern’s effect on temperature lead to a corresponding change in thermoregulatory behavior. Thus, our results reinforce the importance of both behavior and color pattern for the function of aposematic signals not only in the context of predation where they are typically studied but also in a thermoregulatory context.

## FUNDING

The work was supported by a Graduate Research Opportunities Worldwide supplement to National Science Foundation Graduate Research Fellowship Program (award number DGE-1143953) and the Academy of Finland (grant # 293513).

We would like to thank Helinä Nisu for help with bird rearing and Tuuli Salmi for assistance with the mealworm experiments. Aimee Deconinck, Geoffrey Legault, Jee Yun Lee, Juan Galarza, Emily Burdfield-Steel, Sara Calhin, and two anonymous reviewers provided valuable feedback on the manuscript. Research using wild *P. major* was conducted with permission of Southwest Finland Centre for Economic Development, Transport and Environment (VARELY/294/2015) and license from the National Animal Experiment Board (ESAVI/9114/04.10.07/2014). 

Author’s contributions: M.E.N. and J.M. designed the studies. M.E.N. conducted the experiments and analyzed the data. M.E.N. led the writing of the manuscript. M.E.N. and J.M. edited the manuscript and approved the final version.

Data accessibility: Analyses reported in this article can be reproduced using the data provided by Nielsen and Mappes (2020).

## Supplementary Material

araa048_suppl_supplementary_Materials

## References

[CIT0001] Addo-Bediako A, Chown SL, Gaston KJ 2000 Thermal tolerance, climatic variability and latitude. Proc Biol Sci. 267:739–745. 10819141 10.1098/rspb.2000.1065 PMC1690610

[CIT0002] Arenas LM, Walter D, Stevens M 2015 Signal honesty and predation risk among a closely related group of aposematic species. Sci Rep. 5:11021. 26046332 10.1038/srep11021 PMC4457162

[CIT0003] Aronsson M, Gamberale-Stille G 2009 Importance of internal pattern contrast and contrast against the background in aposematic signals. Behav Ecol. 20:1356–1362.

[CIT0004] Aronsson M, Gamberale-Stille G 2013 Evidence of signaling benefits to contrasting internal color boundaries in warning coloration. Behav Ecol. 24:349–354.

[CIT0005] Barnett JB, Scott-Samuel NE, Cuthill IC 2016 Aposematism: balancing salience and camouflage. Biol Lett. 12:20160335. 27484645 10.1098/rsbl.2016.0335 PMC5014027

[CIT0006] Bates D, Maechler M, Bolker B, Walker S 2015 Fitting linear mixed-effects models using lme4. J Stat Softw. 67:1–48. doi: 10.18637/jss.v067.i01.

[CIT0007] Bennett VA, Kukal O, Lee RE 1999 Metabolic opportunists: feeding and temperature influence the rate and pattern of respiration in the high arctic woollybear caterpillar *Gynaephora groenlandica* (Lymantriidae). J Exp Biol. 202:47–53. 9841894 10.1242/jeb.202.1.47

[CIT0008] Birkemoe T, Leinaas HP 2000 Effects of temperature on the development of an Arctic Collembola *(Hypogastrura tullbergi)*. Funct Ecol. 14:693–700. doi: 10.1046/j.1365-2435.2000.00478.x.

[CIT0009] Briolat ES, Burdfield-Steel ER, Paul SC, Rönkä KH, Seymoure BM, Stankowich T, Stuckert AMM 2019 Diversity in warning coloration: selective paradox or the norm? Biol Rev Camb Philos Soc. 94: 388–414. 30152037 10.1111/brv.12460 PMC6446817

[CIT0010] Clusella Trullas S, van Wyk J-H, Spotila JR 2007 Thermal melanism in ectotherms. J Therm Biol. 32:235–245.

[CIT0011] Endler JA, Mappes J 2004 Predator mixes and the conspicuousness of aposematic signals. Am Nat. 163:532–547. 15122501 10.1086/382662

[CIT0012] Galarza JA, Dhaygude K, Ghaedi B, Suisto K, Valkonen J, Mappes J 2019 Evaluating responses to temperature during pre-metamorphosis and carry-over effects at post-metamorphosis in the wood tiger moth (*Arctia plantaginis*). Philos Trans R Soc Lond B Biol Sci. 374:20190295. 31438813 10.1098/rstb.2019.0295 PMC6711291

[CIT0013] Gosler A, Clement P 2007 Family Paridae (tits and chickadees). In del Hoyo J, Elliott A, Christie D, editors. Handbook of the birds of the World. Picathartes to tits and chickadees. Vol. 12 Barcelona (Spain): Lynx Edicions p. 662–709.

[CIT0014] Grant JB 2007 Ontogenetic colour change and the evolution of aposematism: a case study in panic moth caterpillars. J Anim Ecol. 76:439–447. 17439461 10.1111/j.1365-2656.2007.01216.x

[CIT0015] Guilford T. 1986 How do “warning colours” work? Conspicuousness may reduce recognition errors in experienced predators. Anim Behav. 34:286–288.

[CIT0016] Hegna RH, Galarza JA, Mappes J 2015 Global phylogeography and geographical variation in warning coloration of the wood tiger moth *(Parasemia plantaginis)*. J Biogeogr. 42:1469–1481.

[CIT0017] Hegna RH, Nokelainen O, Hegna JR, Mappes J 2013 To quiver or to shiver: increased melanization benefits thermoregulation, but reduces warning signal efficacy in the wood tiger moth. Proc Biol Sci. 280:20122812. 23363631 10.1098/rspb.2012.2812 PMC3574392

[CIT0018] Huey RB, Kingsolver JG 1989 Evolution of thermal sensitivity of ectotherm performance. Trends Ecol Evol. 4:131–135. 21227334 10.1016/0169-5347(89)90211-5

[CIT0019] Joron M, Mallet JL 1998 Diversity in mimicry: paradox or paradigm? Trends Ecol Evol. 13:461–466. 21238394 10.1016/s0169-5347(98)01483-9

[CIT0020] Karpestam E, Wennersten L, Forsman A 2012 Matching habitat choice by experimentally mismatched phenotypes. Evol Ecol. 27:893–907.

[CIT0021] Kingsolver JG 1987 Evolution and coadaptation of thermoregulatory behavior and wing pigmentation pattern in pierid butterflies. Evolution. 41:472–490. 28563799 10.1111/j.1558-5646.1987.tb05819.x

[CIT0022] Kukal O, Heinrich B, Duman JG 1998 Behavioural thermoregulation in the freeze-tolerant Arctic caterpillar, *Gynaephora groenlandica*. J Exp Biol. 138:181–193.

[CIT0023] Lenth R 2018 emmeans: estimated marginal means, aka least-squares means. R package version 1.3.1. Available from: https://CRAN.R-project.org/package=emmeans (accessed 20 December 2018).

[CIT0024] Lindstedt C, Eager H, Ihalainen E, Kahilainen A, Stevens M, Mappes J 2011 Direction and strength of selection by predators for the color of the aposematic wood tiger moth. Behav Ecol. 22:580–587.

[CIT0025] Lindstedt C, Lindström L, Mappes J 2008 Hairiness and warning colours as components of antipredator defence: additive or interactive benefits? Anim Behav. 75:1703–1713.

[CIT0026] Lindstedt C, Lindström L, Mappes J 2009 Thermoregulation constrains effective warning signal expression. Evolution. 63:469–478. 19154362 10.1111/j.1558-5646.2008.00561.x

[CIT0027] Lindstedt C, Morehouse N, Pakkanen H, Casas J, Christedes J-P, Kemppainen K, Lindström L, Mappes J 2010 Characterizing the pigment composition of a variable warning signal of *Parasemia plantaginis* larvae. Funct Ecol. 24:759–766.

[CIT0028] Lindstedt C, Schroderus E, Lindström L, Mappes T, Mappes J 2016 Evolutionary constraints of warning signals: a genetic trade-off between the efficacy of larval and adult warning coloration can maintain variation in signal expression. Evolution. 70:2562–2572. 27624666 10.1111/evo.13066

[CIT0029] Mappes J, Kokko H, Ojala K, Lindström L 2014 Seasonal changes in predator community switch the direction of selection for prey defences. Nat Commun. 5:5016. 25247589 10.1038/ncomms6016 PMC4199109

[CIT0030] Mappes J, Marples N, Endler JA 2005 The complex business of survival by aposematism. Trends Ecol Evol. 20:598–603. 16701442 10.1016/j.tree.2005.07.011

[CIT0031] Muñoz MM, Stimola MA, Algar AC, Conover A, Rodriguez AJ, Landestoy MA, Bakken GS, Losos JB 2014 Evolutionary stasis and lability in thermal physiology in a group of tropical lizards. Proc Biol Sci. 281:20132433. 24430845 10.1098/rspb.2013.2433 PMC3906933

[CIT0032a] Nice CC, Fordyce JA. 2006 How caterpillars avoid overheating: behavioral and phenotypic plasticity of pipevine swallowtail larvae. Oecologia. 146:541–548. 16133191 10.1007/s00442-005-0229-7

[CIT0032] Nielsen ME, Levin E, Davidowitz G, Papaj DR 2018 Colour plasticity alters thermoregulatory behaviour in *Battus philenor* caterpillars by modifying the cue received. Anim Behav. 140:93–98.

[CIT0033] Nielsen ME, Mappes J 2020 Data from: Out in the open: behavior’s effect on predation-risk and thermoregulation by aposematic caterpillars. Behav Ecol. doi: 10.5061/dryad.95x69p8gk. PMC7390994 32760178

[CIT0034] Nielsen ME, Papaj DR 2017 Why have multiple plastic responses? Interactions between color change and heat avoidance behavior in *Battus philenor* larvae. Am Nat. 189:657–666. 28514633 10.1086/691536

[CIT0035] Nokelainen O, Hegna RH, Reudler JH, Lindstedt C, Mappes J 2012 Trade-off between warning signal efficacy and mating success in the wood tiger moth. Proc Biol Sci. 279:257–265. 21653589 10.1098/rspb.2011.0880 PMC3223681

[CIT0036] Nokelainen O, Valkonen J, Lindstedt C, Mappes J 2014 Changes in predator community structure shifts the efficacy of two warning signals in Arctiid moths. J Anim Ecol. 83:598–605. 24164666 10.1111/1365-2656.12169

[CIT0037] Ojala K, Lindström L, Mappes J 2007 Life-history constraints and warning signal expression in an arctiid moth. Funct Ecol. 21:1162–1167.

[CIT0038] Pinheiro CEG 1996 Palatability and escaping ability in Neotropical butterflies: test with wild kingbirds. Biol J Linn Soc. 59:351–365.

[CIT0039] Pinheiro CEG 2007 Asynchrony in daily activity patterns of butterfly models and mimics. J Trop Biol. 23:119–123.

[CIT0040] Pinheiro J, Bates D, DebRoy S, Sarkar D; R Core Team 2018 nlme: linear and nonlinear mixed effects models. R package version 3.1–131.1 Available from: https://CRAN.R-project.org/package=nlme (accessed 20 February 2018).

[CIT0041] Prudic KL, Skemp AK, Papaj DR 2006 Aposematic coloration, luminance contrast, and the benefits of conspicuousness. Behav Ecol. 18:41–46.

[CIT0042] R Core Team 2018 R: a language and environment for statistical computing. Vienna (Austria): R Foundation for Statistical Computing Available from: https://www.R-project.org/ (accessed 1 May 2017).

[CIT0043] Rößler DC, Lötters S, Mappes J, Valkonen JK, Menin M, Lima AP, Pröhl H 2019 Sole coloration as an unusual aposematic signal in a Neotropical toad. Sci Rep. 9:1128. doi:10.1038/s41598-018-37705-1. 30718568 PMC6362010

[CIT0044] Rönkä K, Mappes J, Kaila L, Wahlberg N 2016 Putting *Parasemia* in its phylogenetic place: a molecular analysis of the subtribe Arctiina (Lepidoptera). Syst Entomol. 41:844–853.

[CIT0045] Rudh A, Breed MF, Qvarnström A 2013 Does aggression and explorative behaviour decrease with lost warning coloration? Biol J Linn Soc 108:116–126. doi: 10.1111/j.1095-8312.2012.02006.x.

[CIT0046] Schneider CA, Rasband WS, Eliceiri KW 2012 NIH image to ImageJ: 25 years of image analysis. Nat Methods. 9:671–675. 22930834 10.1038/nmeth.2089 PMC5554542

[CIT0047] Sherman PW, Watt WB 1973 The thermal ecology of some Colias butterfly larvae. J Comp Physiol. 83:25–40.

[CIT0048] Speed MP, Brockhurst MA, Ruxton GD 2010 The dual benefits of aposematism: predator avoidance and enhanced resource collection. Evolution. 64:1622–1633. 20050915 10.1111/j.1558-5646.2009.00931.x

[CIT0049] Speed MP, Ruxton GD 2005 Aposematism: what should our starting point be? Proc R Soc B. 272:431–438. 10.1098/rspb.2004.2968 PMC1634992 15734698

[CIT0050] Stevens M, Ruxton GD 2012 Linking the evolution and form of warning coloration in nature. Proc Biol Sci. 279:417–426. 22113031 10.1098/rspb.2011.1932 PMC3234570

[CIT0051] Stevenson RD 1985 The relative importance of behavioral and physiological adjustments controlling body temperature in terrestrial ectotherms. Am Nat. 126:362–386.

[CIT0052] Sunday JM, Bates AE, Kearney MR, Colwell RK, Dulvy NK, Longino JT, Huey RB 2014 Thermal-safety margins and the necessity of thermoregulatory behavior across latitude and elevation. Proc Natl Acad Sci USA. 111:5610–5615. 24616528 10.1073/pnas.1316145111 PMC3992687

[CIT0053] Tabadkani SM, Nozari J 2014 Relaxed predation hinders development of anti-predator behaviors in an aposematic beetle. Entomol Exp Appl. 153:199–206. doi: 10.1111/eea.12241.

[CIT0054] Valkonen JK, Nokelainen O, Jokimäki M, Kuusinen E, Paloranta M, Peura M, Mappes J 2014 From deception to frankness: benefits of ontogenetic shift in the anti-predator strategy of alder moth *Acronicta alni* larvae. Curr Zool. 60:114–122. doi:10.1093/czoolo/60.1.114.

[CIT0055] Willink B, Brenes-Mora E, Bolaños F, Pröhl H 2013 Not everything is black and white: color and behavioral variation reveal a continuum between cryptic and aposematic strategies in a polymorphic poison frog. Evolution. 67:2783–2794. 24094333 10.1111/evo.12153

