# Supplementary material for: Out in the open: behavior’s effect on predation risk and thermoregulation by aposematic caterpillars
Source: Behav Ecol. 2020 May 20;31(4):1031–9. doi: 10.1093/beheco/araa048 (PMC7390994; doi:10.1093/beheco/araa048)
Supplement: araa048_suppl_supplementary_Materials [file araa048_suppl_supplementary_materials.docx]

Supplemental methods

*Assessment of behavior in short term behavioral exposure experiment*

We used a threshold of 20% shaded when a caterpillar stopped moving to identify thermoregulatory behavior. To increase our confidence that this indeed represented heat-avoidance, we continued the trial until either the caterpillar stopped with at least 50% of its body shaded or 20 minutes had elapsed. During this additional time, two caterpillars moved to and stopped in the open which suggested they were not seeking a cooler location during their original movement and they were thus excluded from the final analysis. In the majority of trials (28 of 45), the 20% and 50% thresholds were met at the same time (i.e. the first time they stopped they were at least 50% shaded), and results were qualitatively similar if data from the 50% shade threshold was used instead of 20%. If a caterpillar fell off the side of the pot at any point during the trial, we immediately replaced it. If they resumed movement within 15 seconds, we considered the movement a continuation of the previous movement, otherwise, we excluded the caterpillar from analysis (7 caterpillars fell off, and 3 were excluded in this way). Due to measurement errors, we had to exclude one additional caterpillar from analysis for ambient temperature, resulting in final sample sizes of 45, 44, and 45 for time, ambient temperature, and body temperature respectively.

*Estimating temperature of cardboard background*

To the effect of the shading treatment on the temperature of the cardboard temperature, we estimated its temperature directly in FLIR Tools (v6.3). We made one measurement for each image (four total, each one quarter of the caterpillar array containing 12 caterpillars) by placing a circle in the center of the image, specifically in the middle row between the second and third caterpillars (Figure S1). FLIR Tools calculated the mean temperature of this circle, based on a provided emissivity of 0.74 and reflected temperature of 3.4°C. Emissivity of cardboard was estimated in the lab based on comparison to tape of a known emissivity. Reflected temperature was estimated based on weather conditions during the trial (emissivity of sky = 0.86, cloud cover = 0.5, air temperature at ground level = 14°C).

Figure S1. Example image of measurement of temperature of cardboard background in FLIR Tools. The circle is the measured area (triangles identifying points of min (red) and max (blue) temperatures). Bright white (high temperature) objects are the caterpillars. This image is of the shaded treatment.

*Predation trial boxes*

Experiments with *Parus major* took place in plywood boxes (50 cm x 50 cm x 67 cm). Boxes were empty except for a wooden horizontal dowel as a perch and a fluorescent light (26 W, Repti Glo 5.0 UVB Compact light bulbs). During trials, the boxes also contained a water bowl. The front wall of each box was a one-way mirror with a black plastic trash bag covering the outside to further obscure any light from outside the box. This set up allowed observation from an otherwise dark room during the trial while minimizing disturbance to the bird. The one-way mirror could also be raised to allow the array of caterpillars to be placed in the box. In additional to the single observer, trials were recorded using a digital camera to allow for verification of observations.
